# When Happiness is Both Joy and Purpose: The Complexity of the Pursuit of Happiness and Well-Being is Related to Actual Well-Being

**DOI:** 10.1007/s10902-022-00541-2

**Published:** 2022-06-10

**Authors:** Julia Krasko, Sabrina Intelisano, Maike Luhmann

**Affiliations:** Department of Psychology, Ruhr University Bochum, Bochum, Germany

**Keywords:** Definitions of happiness, Lay definitions, Happiness-related intentions, Well-being, Happiness, Test construction

## Abstract

People differ in how they define and pursue happiness and well-being (HWB). Previous studies suggested that the best way to achieve a high level of well-being might be to pursue different facets of HWB simultaneously. We expand on this idea and introduce the concept of *complexity of HWB definitions* to describe how many HWB definitions people *endorse* simultaneously, and the *complexity of HWB-related intentions* to describe how many unique facets of HWB people *intend* to pursue in everyday life. To operationalize these novel concepts, we developed two parallel measures that integrate psychological and philosophical definitions of HWB. In two independent studies (total *N* = 542), exploratory and confirmatory factor analyses revealed eight reliable and valid factors for both parallel scales: absence of negativity, positive attitude, tranquility, personal development, luck, joy and desires, purpose, and belonging. Complexity of HWB-related intentions was positively associated with all facets of well-being, whereas complexity of HWB definitions was only positively associated with some facets of well-being. HWB-related intentions and their complexity emerged as more important for the experience of well-being than HWB definitions and their complexity. These studies highlight the importance of a multifaceted conceptualization of HWB when investigating how the pursuit of HWB is related to actual levels of well-being.

What is happiness and what is the best way to be happy? An overwhelming number of opinions and answers exist to this very old question. Happiness and well-being (HWB) have been described in many different ways in the academic literature (Intelisano et al., 2020; Tov, 2018) and by lay people (Delle Fave et al., 2011; Pflug, 2009). We use the term *HWB definitions* to refer to different ways HWB is conceptualized—in the academic literature or by lay people—and we use the term *lay definitions* to specifically refer to lay people’s HWB definitions. In the philosophical and psychological literature, the distinction between the terms happiness and well-being is not always clear and the terms are either often used synonymously or with differing meanings (Haybron, 2001, 2007; Intelisano et al., 2020). For example, in philosophy, theories of well-being refer to normative value concepts like prominent eudaimonic theories whereas theories of happiness are rather descriptive and refer to valued subjective psychological states (Haybron, 2007; Intelisano et al., 2020; Waterman, 2008). In psychology, however, concepts like psychological functioning and eudaimonic well-being are treated as subjective conditions albeit being related to normative eudaimonic theories in philosophy (Intelisano et al., 2020; Ryff, 1989b; Waterman, 2008). This reflects an inconsistent use of the term well-being in the literature. Further, although lay people seem to generally agree that happiness and well-being are two distinct concepts (Jongbloed & Andres, 2015), they frequently attribute a broad range of concepts to the term happiness, including concepts that some scholars in psychology or philosophy would attribute to theories of well-being (see Delle Fave et al., 2016; Jongbloed & Andres, 2015; McMahan & Estes, 2011b; Oishi et al., 2013). Although we were particularly interested in lay people’s definitions of happiness, we therefore also take concepts into account that some scholars would attribute to theories of well-being. For this reason, we frequently refer to both happiness and well-being definitions in our manuscript.

There is no one single way to actively pursue happiness. Rather, it seems to matter how people define HWB for themselves and which specific facet of HWB they strive for (McMahan & Estes, 2011a; Steger et al., 2008). For example, in one study, defining happiness in eudaimonic terms was higher and more robustly associated with well-being than defining happiness in hedonic terms (McMahan & Estes, 2011a). Other studies suggest that the best way to achieve a high level of well-being might be to not focus on one particular facet of HWB (e.g., either eudaimonic or hedonic HWB), but rather to pursue different facets of HWB simultaneously (Henderson & Knight, 2012; Huta & Ryan, 2010; Sirgy & Wu, 2009). In the present paper, we expand on this idea and introduce the concept of *complexity* of HWB definitions to describe how many HWB definitions people endorse simultaneously. The complexity of HWB definitions varies on a continuum from simple (i.e., endorsing only one or few HWB definitions) to complex (i.e., endorsing multiple HWB definitions simultaneously).

To understand how lay people actively pursue happiness, it is not sufficient to only look at which HWB definitions they endorse. Instead, we additionally need to examine to what extent people intend to pursue different facets of HWB in everyday life. Just like people might endorse few or many HWB definitions (definitions-complexity), they might also intend to pursue few or many different facets of HWB in everyday life. We label this concept as the complexity of HWB-related intentions (intentions-complexity). Intentions-complexity varies on a continuum from focused (i.e., pursuing one or few facets of HWB) to diffuse (i.e., pursuing several facets of HWB simultaneously).

The primary goal of this paper was to examine the relationships of HWB definitions-complexity and HWB-related intentions-complexity with actual levels of well-being. To examine these relationships, measures are needed that assess multiple different HWB lay definitions and HWB-related intentions. As we elaborate below, existing measures assessing these or related constructs (Huta & Ryan, 2010; McMahan & Estes, 2011b; Peterson et al., 2005) are not sufficient for our purposes. A secondary goal was therefore to develop novel measures of HWB lay definitions and HWB-related intentions that allow us to empirically investigate the complexity of HWB definitions and HWB-related intentions.

## The Complexity of Happiness Definitions

Is the complexity of HWB definitions related to actual levels of well-being? Similar questions have been discussed and investigated before (Grimm et al., 2015; Huta & Ryan, 2010; Sirgy & Wu, 2009). For example, Sirgy and Wu (2009) proposed that people who have a balance between a pleasant life, an engaged life, and a meaningful life experience higher levels of well-being than people who have an imbalance. In line with this proposal, studies showed that pursuing different facets of HWB simultaneously is associated with higher levels of well-being than focusing on only one facet (Grimm et al., 2015; Huta & Ryan, 2010). Accordingly, a balanced satisfaction of basic psychological needs contributes to well-being over and above the aggregated need fulfillment (Milyavskaya et al., 2009; Sheldon & Niemiec, 2006). These basic psychological needs have been described in the Self-Determination Theory and are related to eudaimonic HWB definitions (Ryan et al., 2008).

In line with this literature, we expect people with complex HWB definitions to experience higher levels of well-being than people with simple HWB definitions. Possible explanations for this link can be summarized as follows: (1) People with complex HWB definitions might be better at spreading their resources across different life domains, whereas people with simple HWB definitions might invest their resources only in few life domains. The latter can lead to shortcomings in other life domains, as well as negative outcomes like stress or role conflict (Sheldon & Niemiec, 2006; Sirgy & Wu, 2009). (2) The successful pursuit of different facets of HWB contributes to the overall level of well-being by affecting different emotional experiences and serving different human needs (Keyes et al., 2002; Sirgy & Wu, 2009). (3) When specific facets of HWB cannot be reached at certain times, other facets of HWB might compensate or buffer an overall negative effect on well-being (Keyes et al., 2002; Sirgy & Wu, 2009). (4) Positive spillover effects from one facet of HWB to another might contribute to a higher overall level of well-being (Sumer & Knight, 2001). For example, hedonic pleasure can result from eudaimonic action (Kashdan et al., 2008).

## From Definitions to Intentions

According to the previous section, people with complex HWB definitions need to live in consistency with their definitions to experience higher levels of well-being. However, people do not always live and act in consistency with their beliefs. For example, someone might agree that positive relationships are an important facet of happiness but have no intentions to improve this facet actively. One possible reason for this discrepancy could be individual beliefs about the (lack of) controllability of happiness (Passmore et al., 2018; Titova & Sheldon, 2019). We propose that how people describe HWB is related but not identical to their *intentions* to pursue specific facets of HWB in everyday life. Further, we expect people with diffuse HWB-related intentions (i.e., pursuing several facets of HWB simultaneously) to experience higher levels of well-being than people with focused HWB-related intentions (i.e., pursuing one or few facets of HWB).

## Measuring Happiness Definitions and Intentions to Pursue Happiness

To examine the relationships of HWB definitions-complexity and HWB-related intentions-complexity with actual levels of well-being, a measure is needed that assesses multiple different HWB lay definitions and HWB-related intentions. Existing measures of these constructs or related constructs (Huta & Ryan, 2010; McMahan & Estes, 2011b; Peterson et al., 2005) cover only a few prominent HWB definitions. For example, the Beliefs About Well-Being Scale (McMahan & Estes, 2011b) measures only four dimensions: experience of pleasure, avoidance of negative experience, self-development, and contribution to others. Consequently, existing measures do not allow us to assess the complexity of HWB definitions and of HWB-related intentions appropriately. Furthermore, existing measures either do not distinguish between HWB-related definitions and intentions (Peterson et al., 2005) or focus on only one of these aspects (Huta & Ryan, 2010; McMahan & Estes, 2011b).

We therefore developed two parallel measures of HWB lay definitions and HWB-related intentions that cover a broader range of different HWB definitions in order to measure HWB definitions-complexity and HWB intentions-complexity. To construct these scales, we needed to decide which of the many existing HWB definitions should be considered when creating an item pool. For this purpose, we reviewed studies on lay definitions of HWB. Some of these studies used unidimensional (Joshanloo, 2019) or multidimensional (McMahan & Estes, 2011b; Peterson et al., 2005) scales. Many of these scales measure aspects of both hedonic and eudaimonic HWB definitions, which are present in lay people’s HWB definitions across cultures (Delle Fave et al., 2011, 2016; Lu & Gilmour, 2004; Pflug, 2009). Hedonic definitions describe HWB as the experience of pleasure and other positive emotions, positive cognitive evaluations of one’s life, and the absence of negative emotions or experiences. Eudaimonic definitions describe HWB as the fulfillment of one’s inner potential, self-development, virtue, meaning, and autonomy (Henderson & Knight, 2012; Huta & Waterman, 2014). Other prominent HWB definitions refer to different aspects of social relationships such as interpersonal connectedness or feelings of acceptance (Keyes, 1998; Uchida & Ogihara, 2012).

Qualitative studies revealed additional HWB definitions that lay people view as important. These studies investigated free-response associations with happiness (Delle Fave et al., 2016; Lu & Gilmour, 2004; Pflug, 2009) or language use related to HWB (Lomas, 2016; Oishi et al., 2013). For example, some people consider HWB as favorable external circumstances that cannot be controlled by an individual, like good luck or fortune (Oishi et al., 2013; Pflug, 2009). Pflug's (2009) investigation of Germans and South Africans revealed that some people describe happiness with statements like “finding a few euros without having expected it” (p. 558), which appears to refer to luck. Such statements might be caused by the fact that in Germany the term “Glück” refers to both happiness and luck. However, Oishi et al. (2013) showed that luck and fortune were present in dictionary definitions of happiness in many nations. Historically, luck was a dominant aspect in HWB definitions (Oishi et al., 2013) and many words that refer to happiness states are etymologically derived from luck (Lomas, 2016; McMahon, 2004). In sum, people seem to agree that “Happiness […] is what happens to us” (McMahon, 2004, p. 8) and that this is at least to some extent dependent on random and uncontrollable external factors. Although scales exist to measure the belief in luck (Darke & Freedman, 1997), luck has been ignored in scales of HWB definitions. One exception is Joshanloo (2017), who investigated the lay belief that happiness depends on factors outside of humans' control, which seems to be related to the concept of luck.

Other less frequently considered HWB definitions cover positive affective states that are characterized by low levels of arousal, like calmness or tranquility (Berenbaum et al., 2019; Delle Fave et al., 2016; Uchida & Ogihara, 2012). For example, Delle Fave et al. (2016) described inner harmony as an umbrella term for such low-arousal affective states. Other examples include research on tranquility, which describes a state of pleasant inactivity and peace with one’s current status (Berenbaum et al., 2019; Ellsworth & Smith, 1988). People from East-Asian cultures are supposed to pursue low-arousal positive affective states. In contrast, people from Western cultures are supposed to pursue high-arousal positive affective states like joy or excitement (Uchida & Ogihara, 2012). However, qualitative studies revealed that inner harmony was also one of the most cited HWB definition among Western cultures (Delle Fave et al., 2016). Although low-arousal positive affective states appear to be important for lay people, no measure of HWB definitions had previously included such states.

Of course, many other not yet mentioned HWB definitions have previously been described. Examples include optimism and an overall positive attitude towards life (Delle Fave et al., 2016; Haybron, 2005, 2007) or physical safety and material well-being (Furnham & Cheng, 2000; Lomas, 2016). We aimed to develop measures that allow us to distinguish several different HWB definitions while it should also have a pragmatic length. Hence, we did not aspire to consider all possible HWB definitions that have ever been described but instead restricted the scale to prominent HWB definitions in the philosophical and psychological HWB literature and for which past studies have shown that they are also relevant for lay people.

## The Present Paper

The main goal of the present paper was to investigate the relationships of the complexity of HWB definitions and of HWB-related intentions with actual levels of well-being. In two independent studies, we developed the CoDI Scales, two novel scales measuring HWB definitions and definitions-complexity (Studies 1 and 2) as well as HWB-related intentions and intentions-complexity (Study 2). Further, we investigated the reliability and validity of the scales (Study 2). The CoDI Scales were then used to examine our main research question (Study 2).

## Study 1

In Study 1, we developed a novel measure of lay definitions of HWB that covers a broader range of definitions than previous scales and allows us to operationalize the complexity of people’s HWB definitions.

### Method

#### Participants

The study was preregistered before the data collection started.^1^ Data collection was approved by the ethics committee of the Department of Psychology, Ruhr University Bochum. Data collection was conducted in May 2017 among a German student sample using the online survey tool Qualtrics. Participants received course credit and could participate in a lottery of Amazon vouchers worth 800€ in total. We aimed to collect data from 200–300 participants since it has been shown that for stable estimates of correlations the sample size should approach 250 (Schönbrodt & Perugini, 2013). We collected responses from 278 participants. We excluded seven participants due to an item for the self-assessment of data quality, six participants due to failed responses on two instructed-response items, and two participants with missing values on at least one item of the new measure. After exclusion, the final sample size was *N* = 263. The age of the participants ranged from 18 to 68 (*M* = 24.49, *SD* = 5.19), 60.3% were female, 95.0% not married, and 59.5% in a relationship.

#### Measures and Procedure

Following common recommendations for test construction (e.g., Downing, 2006), we initially developed a comprehensive pool of 270 items for HWB definitions. For item development, we systematically considered HWB definitions that were listed in an integrative review of philosophical and psychological definitions of HWB (Intelisano et al., 2020). This review included prominent HWB definitions in the philosophical and psychological literature as well as studies of lay definitions of HWB (e.g., McMahan & Estes, 2011b). Because of the importance of uncontrollable favorable external circumstances (i.e., luck) in lay definitions of HWB (Delle Fave et al., 2016; Oishi et al., 2013; Pflug, 2009), we additionally included items to capture this construct. For the specific item wordings, we were inspired by established measures related to these HWB definitions (e.g., Hitokoto & Uchida, 2015; Peterson et al., 2005; Ryff, 1989a) and a review of dictionary definitions of happiness (Oishi et al., 2013). Because of substantial overlaps among the included HWB definitions, the initial item pool had a large number of redundant items. We reduced the item pool to 110 items after peer feedback. The peers were instructed to focus on item redundancies and item quality according to common recommendations for item wordings. The items were phrased as a completion of a specific item stem (“*For me personally, happiness means…*”) (Table 1). Further, the items were phrased in a general manner such that they assess whether people generally endorse specific HWB definitions regardless of whether they actively pursue specific facets of HWB.

**Table 1:** Overview of characteristics of the parallel scales

	Item stem	Example Item	Wording
HWB definitions	For me personally, happiness means …	…calmness and tranquility	General
HWB-related intentions	In daily life, I try …	…to be calm and tranquil	Subjective

HWB = happiness and well-being

Item responses were averaged within subscales to create scores. The complexity of HWB definitions was calculated by averaging the subscales representing different HWB definitions.^2^ A strong agreement with many items for HWB definitions would lead to high scores for the subscales, which in turn would result in a high complexity score. In contrast, a strong agreement with only one or few subscales and a low agreement with the other subscales would result in a low complexity score.

Response tendencies like acquiescence are a common issue in research that depends on self-reports (Danner et al., 2015; Soto et al., 2008; Wetzel et al., 2013). The above-described approach to compute complexity would not only result in a high complexity score when participants endorse many HWB definitions but also when they tend to agree on items irrespective of the specific content. To ensure that the results of this study and particularly the scores for complexity are not confounded with response tendencies, we included eight items that were phrased as opposite statements of items for HWB definitions. For example, for the item “…not to experience negative emotions” the corresponding item “…to also experience negative emotions once in a while” was included. We calculated an acquiescence score using responses to these pairs of items representing opposing statements (Danner et al., 2015; Ferrando et al., 2004; Soto et al., 2008). For the acquiescence score, we considered only item pairs with sufficient Pearson’s correlations (*r* < –.30). The score was calculated by averaging these items such that the resulting score represents the typical content-independent response, which should be the mid-point of the scale (3.5) for participants without any response tendencies. We subtracted the value 3.5 from each score so that the resulting score represents deviations from the mid-point of the scale. Item responses were adjusted for response tendencies by subtracting this acquiescence score from each item response. Previous studies showed that efforts to control for response tendencies improved the results (Soto et al., 2008).

Items for HWB definitions and response tendencies were presented in blocks of 20 randomly ordered items. Participants were asked to indicate to what extent they agree with the statements using a Likert scale. We used two different labeling conditions for the response options: For 48.3% of participants, we used an asymmetric scale label with only one level for true rejection (1 = *does not apply*) and five levels indicating different nuances of agreement (from 2 = *applies hardly* to 6 = *applies completely*). For 51.7% of participants, we used scale labels ranging from 0 (*does not apply*) to 5 (*applies completely*) with labels for only the two boundary points. We used these two different conditions to investigate the impact of scale labeling on response tendencies. The acquiescence score did not differ significantly between the two labeling conditions, *F*(1, 261) = 0.003, *p* = .959, Cohen’s *d* = − 0.006.

To control for data quality (see Meade & Craig, 2012), we additionally included two instructed-response items (e.g., “To assess data quality, please choose response option no. 2”), one dichotomous item for self-assessment of data quality (i.e., directly asking the participants whether they answered the survey questions appropriately), and one open question for comments of any kind. Further, we assessed basic demographics such as age and gender.

### Results and Discussion

All analyses were conducted in R (R Core Team, 2020), using the package *psych* (Revelle, 2017). To quantify associations, we used Pearson’s product-moment correlation coefficients. We refer to item responses unadjusted for acquiescence unless stated otherwise. First, we reduced the item pool by omitting poorly performing items. As criteria for item reduction, we considered item difficulties (Lord, 1952; poorly when outside a scale range of 2.5–4.5 on the 1–6 Likert scale), discriminatory power (Jackson, 1970; poorly when < .35), and visual inspections of item-specific response distributions (poorly when visibly skewed). We rated the performances of all items on these criteria. We omitted 29 items with at least three negative ratings on these criteria in total by taking both into account the unadjusted and adjusted item responses. Items with fewer negative ratings remained in the item pool for the next step.

81 items were included in an exploratory factor analysis. The number of factors was determined using parallel analysis (Goretzko et al., 2019; Horn, 1965), which suggested 8 factors with similar patterns for both the unadjusted and adjusted items. We extracted 8 factors using principal axis analysis and Promax rotation. For factor interpretation, we considered items with minimum loadings of .30 on a single factor, which is an appropriate cutoff criterion for this sample size (Field et al., 2012; Stevens, 2002).

Most of the factors were clearly interpretable (online materials Table A^3^). Factor 1 described HWB as the absence of negative affect (*absence of negativity*, e.g., “… to rarely feel bad.”). The view that negative affective states and experiences are an obstacle to happiness is also present in hedonic HWB definitions (Henderson & Knight, 2012; Huta & Waterman, 2014).

Factor 2 referred to a generally positive attitude towards life (*positive attitude*, e.g., “… to cheerfully go through life.”) as well as to low-arousal positive affective states (*tranquility*, e.g., “… a sense of inner peace.”). This factor combines two HWB definitions described in the literature. Our goal was, therefore, to assess them separately in the final version of the measure. Positive attitude describes HWB as a propensity for positive mood, which has previously been described in the literature (Delle Fave et al., 2016; Haybron, 2007). Tranquility refers to pleasant inactivity and peace with one’s current status (Berenbaum et al., 2019; Ellsworth & Smith, 1988) and low-arousal positive affective states described in the literature (Delle Fave et al., 2016; Uchida & Ogihara, 2012).

Factor 3 referred to interest and continuous development in life (*personal development*; e.g., “… to develop beyond oneself.”). Describing HWB as a successful process of self-development and the fulfillment of one’s inner potential is a core facet of most eudaimonic theories (Huta & Waterman, 2014; Intelisano et al., 2020; Ryff & Singer, 2008).

Factor 4 referred to luck and fortune (*luck*; e.g., “… to be favored by luck or fortune.”). Describing HWB as favorable circumstances outside the own control is an ancient view that is still prevalent in many cultures (McMahon, 2004; Oishi et al., 2013; Pflug, 2009).

Factor 5 referred to the experience of pleasure, satisfaction, and the fulfillment of desires (*joy and desires*; e.g., “… the extent to which current preferences are satisfied.”). Describing HWB as the experience of pleasure and satisfaction is related to hedonic HWB definitions (Diener, 1984; Huta & Waterman, 2014) and to philosophical theories that describe HWB as the fulfillment of one’s desires (Intelisano et al., 2020).

Factor 6 referred to having a sense of purpose in life (*purpose*; e.g., “… that the own life has a lasting meaning.”). HWB as the presence of a greater goal in one’s life that directs one’s general decisions has previously also been described by many eudaimonic HWB definitions (Huta & Waterman, 2014; Intelisano et al., 2020; Ryff & Singer, 2008).

Factor 7 referred to closeness and connection to others (*belonging*; e.g., “… to feel related.”). Relational aspects like interpersonal connectedness and social integration have frequently been emphasized in HWB definitions by lay people and scholars (Delle Fave et al., 2011; Keyes, 1998; Uchida & Ogihara, 2012).

Factor 8 was the only factor that could not be clearly interpreted because it included only a few items with weak loadings and double loadings. For this reason, we dropped this factor. Generally, it appears to reflect HWB as intense and transcendent feelings (e.g., “… a short, very intense feeling that the world is entirely good”).

To sum up, some factors reflected HWB definitions that have previously been described in the literature and have been covered by other scales assessing HWB definitions (e.g., the absence of negativity, purpose; McMahan & Estes, 2011b; Peterson et al., 2005). In addition, the exploratory factor analysis revealed factors that have previously not been included in other scales of HWB definitions (e.g., tranquility and luck).

In the next step, we selected between 4 and 6 items per factor to compute scale scores and for further investigations in Study 2. For this item selection, we considered factor loadings, whether relations of factors and items differed between unadjusted and adjusted versions of the items, and how the exclusion would affect the internal consistency of the subscales. In total, 40 items remained. Cronbach’s alpha ranged between α = .68 and α = .84. The correlation between the factors ranged between *r* = .12 (*p* = .043) and *r* = .65 (*p* < .001) (Table 2).

**Table 2:** Descriptive statistics and correlations of Study 1

Variable		Unadjusted			Adjusted			1	2	3	4	5	6	7	8	9	10
		*M*	*SD*	α	*M*	*SD*	α										
1	Positive Attitude	4.52	0.93	.79	4.26	0.88	.77	**.69**	.65	.47	.47	.37	.31	.41	.38	.78	.45
2	Tranquility	4.64	0.94	.78	4.37	0.90	.76	.61	**.70**	.27	.35	.41	.42	.28	.20	.68	.44
3	Joy and Desires	4.65	0.68	.68	4.38	0.79	.76	.48	.30	**.53**	.31	.21	.27	.33	.26	.56	.36
4	Absence of Negativity	3.89	1.06	.82	3.62	0.93	.77	.35	.21	.24	**.75**	.18	.29	.34	.49	.69	.51
5	Personal Development	4.12	0.84	.70	3.86	0.85	.72	.34	.39	.31	.05	**.64**	.48	.27	.12	.57	.41
6	Purpose	4.18	1.02	.75	3.92	1.02	.74	.28	.40	.33	.20	.48	**.75**	.38	.18	.64	.35
7	Belonging	4.51	0.92	.72	4.24	0.97	.74	.40	.28	.43	.27	.31	.41	**.71**	.24	.62	.33
8	Luck	3.75	1.23	.84	3.49	1.25	.84	.38	.20	.33	.47	.16	.20	.29	**.83**	.61	.26
9	Definitions-Complexity	4.28	0.62	.79	4.02	0.60	.79	.74	.65	.65	.56	.58	.64	.66	.64	**.31**	.60
10	Acquiescence	.27	0.72	–	–	–	–	− .34	− .34	− .59	− .19	− .43	− .35	− .43	− .32	− .58	–

Values above the diagonal indicate correlations between variables unadjusted for acquiescence, values below the diagonal indicate correlations between variables adjusted for acquiescence; bold values represent correlations between unadjusted and adjusted variables

The complexity of HWB definitions was approximately normally distributed. Acquiescence was approximately normally distributed and adjusting for response tendencies normalized the distributions of item responses.^4^ Correlations between acquiescence and HWB definitions as well as complexity ranged between medium and large effect sizes (according to Cohen, 1988). This indicates that people who tend to agree on opposite statements also tend to agree on items for HWB definitions and demonstrates the importance of taking response tendencies into account.

## Study 2

The main objective of this paper was to examine the relationships of the complexity of HWB definitions and of HWB-related intentions with actual levels of well-being. To attain this objective, we pursued multiple goals with Study 2: First, we investigated the psychometric properties of the novel measure of HWB definitions and examined whether we could replicate the factor structure found in Study 1 using an independent sample. The results of Study 1 indicated that positive attitude and tranquility might be represented by one single factor. We investigated whether these two definitions could be measured separately.

Second, we developed and evaluated a parallel measure of intentions to pursue specific facets of HWB that should also allow us to assess the complexity of HWB-related intentions. We investigated the psychometric properties of this parallel measure and examined whether we could replicate the factor structure of HWB definitions for HWB-related intentions. We revised the item pool by selecting items for the final version of the scales.

Third, we investigated the nomological net of the novel measures by examining correlations between HWB lay definitions and HWB-related intentions with existing scales, personality, and actual levels of well-being. The direction of expected correlations between the novel measures and other scales were preregistered^5^ and should have an effect size of *r* >|.30| to provide evidence for meaningful associations (medium effect size according to Cohen, 1988). We did not expect that the overall pattern of associations would differ between HWB definitions and HWB-related intentions.

Finally, we investigated associations between HWB definitions-complexity, HWB intentions-complexity, and actual levels of well-being. We expected that definitions-complexity would be positively associated with intentions-complexity. Further, we examined whether having more complex HWB definitions and more diffuse HWB-related intentions is associated with higher levels of well-being, as suggested by previous work on similar questions (Grimm et al., 2015; Huta & Ryan, 2010; Sirgy & Wu, 2009). For this, we examined associations with subjective well-being (Diener, 1984) and psychological well-being (Ryff, 1989c).

### Method

#### Participants

Data collection started in November 2017 among a German sample (55.6% students) using the online survey tool Qualtrics. Data collection was approved by the ethics committee of the Department of Psychology, Ruhr University Bochum. Participants received course credit and could participate in a lottery of Amazon vouchers worth 600€ in total. We aimed to collect data from 200 to 300 participants since it has been shown that for stable estimates of correlations the sample size should approach 250 (Schönbrodt & Perugini, 2013). We collected responses from 334 participants and excluded four cases due to their responses to an item for self-assessment of data quality, 22 cases due to failed responses on two instructed-response items, and four cases with missing values on at least one item of the novel measures. After exclusion, the final sample size was *N* = 279. Participants’ age ranged from 18 to 80 (*M* = 25.88, *SD* = 7.75), 84.6% were female, 88.2% not married, and 64.2% in a relationship.

#### Measures and Procedure

First, we assessed the novel measures and several related scales for validation purposes in a randomized order for each participant. Items within the scales were also randomized for each participant. At the end of the survey, we assessed basic demographics. Unless otherwise stated below, responses were collected using a scale ranging from 1 (*disagree strongly*) to 5 (*agree strongly*) and were averaged within scales to create scores. Descriptive statistics and Cronbach’s alphas are displayed in Table 3 for the novel measures and Table 4 for the other scales.

**Table 3:**
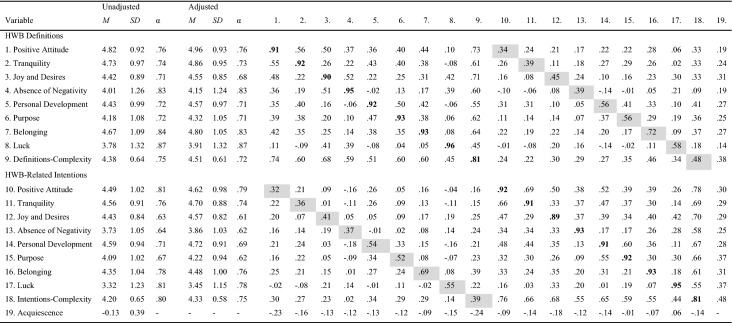
Descriptive statistics and correlations between the CoDI subscales of Study 2

Note. Values above the diagonal indicate correlations between variables unadjusted for acquiescence, values below the diagonal indicate correlations between variables adjusted for acquiescence; bold values represent correlations between unadjusted and adjusted variables; grey shaded values represent correlations between corresponding variables for HWB definitions and HWB-related intentions.

**Table 4:**
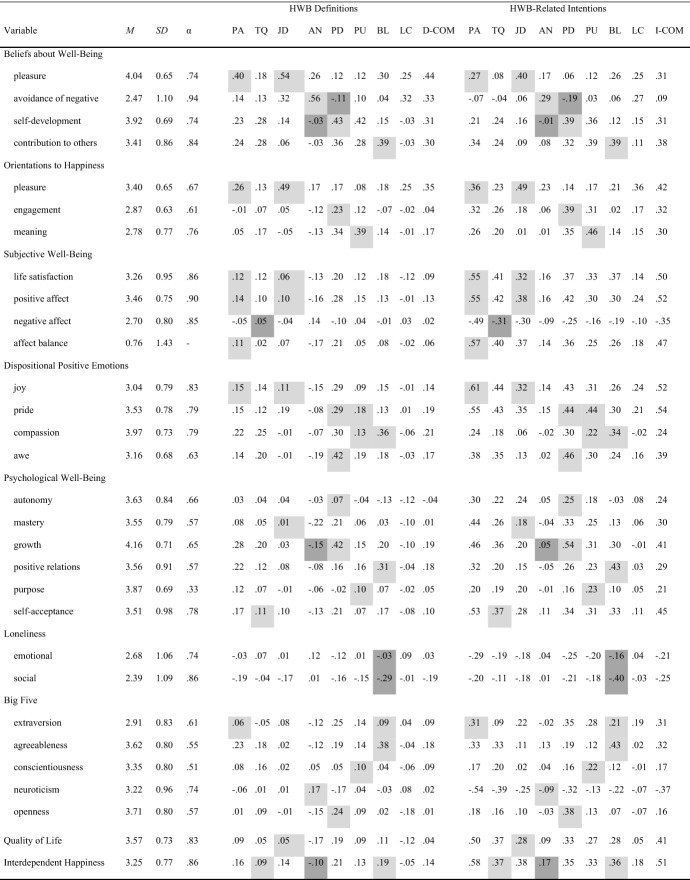
Descriptive statistics and bivariate correlations of the CoDI Scales with related constructs and well-being

Note. HWB = happiness and well-being; PA = positive attitude; TQ = tranquility; JD = joy and desires; AN = absence of negativity; PD = personal development; PU = purpose; BL = belonging; LC = luck; D-COM = definitions-complexity; I-COM = intentions-complexity; light grey = preregistered positive associations; dark grey = preregistered negative associations.

##### HWB Definitions and their Complexity

HWB definitions were assessed with 40 selected items as described in Study 1. Since different conditions of scale labeling revealed no differences in Study 1, we used only the asymmetric scale labels with one level for true rejection (1 = *does not apply*) and five levels indicating different nuances of agreement (from 2 = *applies hardly* to 6 = *applies completely*). Definitions-complexity was computed by averaging the subscale scores representing different HWB definitions as described in Study 1.

##### HWB-Related Intentions and their Complexity

For the parallel measure to assess HWB-related intentions, we created corresponding items for each item of HWB definitions. The items were phrased as a completion of a specific item stem (“*In daily life, I try …*”) (Table 1). Further, the items were phrased in a subjective manner to ensure that they assess whether people intend to pursue specific facets of HWB in everyday life, regardless of whether they generally endorse specific definitions. Responses were collected using the same scale labels as for HWB definitions. Intentions-complexity was calculated in accordance with definitions-complexity by averaging the subscale scores representing different HWB-related intentions.

##### Acquiescence

Similar to Study 1, we included 12 pairs of items with opposite statements to adjust for response tendencies, which were selected in a separate pretest. The items were presented together with items for HWB-related intentions in a randomized order and applied to the same item stem. Seven item pairs had sufficiently strong Pearson’s correlations (*r* < –.30) to be included in the calculation of an acquiescence score. Procedures for calculating acquiescence and adjusting items for HWB definitions and HWB-related intentions were the same as in Study 1.

##### Beliefs About Well-Being

The Beliefs about Well-Being scale by McMahan and Estes (2011a, 2011b) assesses to what extent people endorse four different definitions of happiness, with four items each: experience of pleasure, avoidance of negative experiences, self-development, and contribution to others. We translated this scale into German using the back-translation method (Brislin, 1970).

##### Orientations to Happiness

The Orientations to Happiness scale by Peterson et al., (2005; German version by Ruch et al., 2010) assesses people's orientations to pleasure, engagement, and meaning in life with six items each.

##### Dispositional Positive Emotions

The Dispositional Positive Emotions scales by Shiota et al., (2006; German version by Güsewell & Ruch, 2012) measures seven different dispositions of positive emotions. To keep the survey short, we selected four subscales that were most relevant for validation purposes and that differed the most from other scales assessed: joy (6 items), pride (5 items), compassion (5 items), and awe (6 items).

##### Subjective Well-Being

Subjective well-being covers positive and negative affective experiences and evaluations of one’s life satisfaction (Diener, 1984). Positive and negative affect were assessed with the Scale of Positive and Negative Experience (Diener et al., 2010; German version by Rahm et al., 2017). Participants were asked to indicate how often they felt a certain affective state during the last four weeks. Positive and negative affect were measured with six items each. Further, we computed affect balance by subtracting negative affect from positive affect. To assess people’s evaluations of one’s life satisfaction, we used the 5-item Satisfaction with Life Scale (Diener et al., 1985; German version by Glaesmer et al., 2011).

##### Psychological Well-Being

We used the 18-item version of Ryff 's scales (1989; German version by Risch et al., 2005) to assess six facets of positive psychological functioning with three items each: autonomy, environmental mastery, personal growth, positive relations with others, purpose in life, and self-acceptance.

##### Quality of Life

The EUROHIS-Quality-Of Life-Indices (Power, 2003; German version by Brähler et al., 2007) was used to assess the quality of life with 8 items. Responses were collected using the following five scale labels: very poor/very dissatisfied/quite rarely or never; poor/dissatisfied/rarely; either-or; good/satisfied/often; very good/very satisfied/very often or always.

##### Loneliness

We used the De Jong Gierveld Loneliness scale (Gierveld De Jong & Van Tilburg, 2006; German version by Tesch-Römer et al., 2013) to assess emotional and social loneliness with three items each.

##### Interdependent Happiness

The 9-item Interdependent Happiness scale by Hitokoto and Uchida (2015) assesses an East-Asian HWB definition according to which happiness is based on interpersonal harmony, ordinariness, and quiescence. We translated this scale into German using the back-translation method (Brislin, 1970).

##### Big Five

We used the Big Five Inventory-2 extra-short form (Soto & John, 2016; German version by Danner et al., 2016) to assess extraversion, agreeableness, conscientiousness, neuroticism, and openness with three items each.

Data quality was controlled as described in Study 1. Further, we assessed basic demographics such as age and gender.

### Results

Analyses were conducted in R (R Core Team, 2020), using the packages psych (Revelle, 2017) and lavaan (Rosseel, 2012). All analyses were conducted using item responses that were both unadjusted and adjusted for acquiescence. As in Study 1, acquiescence was approximately normally distributed and adjusting for response tendencies normalized the distributions of item responses.^6^ We refer to item responses unadjusted for acquiescence unless stated otherwise.

#### Factor Structure

The goals of the analyses described in this section were to evaluate the factor structure found in Study 1, to select the final items for the scale by investigating which factor structure performed well for both parallel scales, and to achieve an acceptable model fit. First, we investigated the factor structure of HWB-related intentions using exploratory factor analysis (online materials Table B).^7^ Parallel analysis (Goretzko et al., 2019; Horn, 1965) suggested seven factors that were extracted using principal axis analysis and oblique rotation method. For items representing luck, belonging, and absence of negativity, the factor structure corresponded to the factor structure of HWB definitions in Study 1. Joy and desires also corresponded to the factor structure found in Study 1, although some additional items loaded on this factor. These items were dropped for the final version of the scales. Most items representing positive attitude and tranquility loaded on the same factor as in Study 1. Another factor emerged that was represented by two items for tranquility with considerable double loadings on the combined positive attitude/ tranquility factor. In contrast to HWB definitions in Study 1, items for personal development and purpose loaded on the same factor.

In the next steps, we conducted multiple confirmatory factor analyses. Model fit was interpreted using established fit indices: RMSEA and its 90% confidence interval should be ≤ .05 for a close fit and < .08 for an acceptable fit (Browne & Cudeck, 1992), SRMR should be < .05 for a close fit, and < .08 for an acceptable fit (Hu & Bentler, 1995), and CFI should be > .97 for a close fit and > .95 for an acceptable fit (Bentler, 1990). For comparisons of nested models, we used χ^2^ difference tests. For other model comparisons, we used AIC (Akaike, 1998) and BIC (Schwarz, 1978), where a smaller value indicates a better fit.

We investigated the factor structure described in Study 1 for HWB definitions by evaluating the model fit with the sample of Study 2. The model contained 8 factors measured by four (belonging, luck, purpose), five (positive attitude, tranquility), or six (absence of negativity, joy and desires, personal development) items. The fit indices ranged from not optimal to acceptable (online materials Table B). Applying the same factor structure to HWB-related intentions also revealed a model fit that ranged from not optimal to acceptable.

To develop a parsimonious scale, we excluded items in the next step. For this, we considered empirical criteria like the factor loadings, how the exclusion of single items would affect reliability and fit statistics, and the criteria that were already considered in Study 1. These different criteria revealed conflicting suggestions regarding the best performing items. Furthermore, differences between HWB definitions and HWB-related intentions revealed conflicting suggestions. We aimed to find a factor solution that worked well for both scales since the final measures should be perfectly parallel versions of each other. In such ambivalent situations, we prioritized good empirical performance of items measuring HWB definitions over performance of items measuring HWB-related intentions. In total, we removed 16 items, which considerably improved the model fit for both parallel versions of the measure (HWB definitions: AIC_diff_ = − 13,262.804, BIC_diff_ = − 13,379.003; HWB-related intentions: AIC_diff_ = − 12,721.404, BIC_diff_ = − 12,837.602).

Next, we tested alternative factor structures as suggested by deviations between exploratory factor analyses and theoretical considerations. More precisely, we tested how the model fit changes when positive attitude and tranquility as well as personal development and purpose would be combined in a single factor. We compared models where we combined the related factors with models where the related factors were modeled separately. For these model comparisons, we used both the initial CFA model (before item exclusion) as well as the final models. Combining positive attitude and tranquility decreased the model fit significantly for all four comparisons (HWB definitions initial χ^2^(7) = 152.94, *p* < .001; final χ^2^(7) = 71.292, *p* < .001; HWB-related intentions initial χ^2^(7) = 105.32, *p* < .001; final χ^2^(7) = 42.143, *p* < .001). Combining personal development and purpose also decreased the model fit significantly for all four comparisons (HWB definitions initial χ^2^(7) = 47.173, *p* < .001; final χ^2^(7) = 55.373, *p* < .001; HWB-related intentions initial χ^2^(7) = 34.292, *p* < .001; final χ^2^(7) = 26.588; *p* < .001). These results suggest that it is reasonable to consider positive attitude and tranquility as well as personal development and purpose as separate factors.

In a final step, we improved the fit of the models by allowing pairs of items to correlate. For this, we identified promising correlations using modification indices. We considered only correlations between items within the same factor such that the correlations are theoretically plausible. We allowed correlations between Items 108 and 36, 106 and 86, and 87 and 51 for both HWB definitions and HWB-related intentions. The fit statistics and psychometric properties of these models were good (Table 5).^8^ The results described in this section were mostly consistent between the unadjusted and adjusted versions of the items.

**Table 5:** Standardized factor loadings and fit statistics for the final CFA models

	HWB Definitions								HWB-Related Intentions							
Item No	PA	TQ	JD	AN	PD	PU	BL	LC	PA	TQ	JD	AN	PD	PU	BL	LC
75	.73								.80							
108	.73								.73							
36	.70								.77							
42		.70								.62						
51		.78								.75						
87		.64								.80						
3			.60								.61					
17			.71								.55					
10			.71								.67					
99				.74								.43				
58				.73								.70				
63				.91								.69				
93					.69								.74			
39					.73								.71			
71					.64								.58			
106						.64								.65		
86						.72								.64		
59						.68								.63		
45							.82								.67	
101							.84								.68	
20							.74								.89	
107								.76								.78
81								.86								.82
103								.87								.70
*Fit statistics*																
χ^2^ (df = 224)	384.62; *p* < .001								441.84; *p* < .001							
AIC	19,601.49								19,765.69							
BIC	19,877.46								20,041.66							
CFI	.94								.91							
TLI	.93								.89							
RMSEA [90% CI]	.05 [.04; .06]								.06 [.05; .07]							
SRMR	.06								.06							
*Fit statistics after consideration of correlations within factors*																
χ^2^ (df = 221)	336.29; *p* < .001								418.94; *p* < .001							
AIC	19,559.16								19,748.79							
BIC	19,846.03								20,023.66							
CFI	.96								.92							
TLI	.95								.90							
RMSEA [90% CI]	.04 [.03; .05]								.06 [.05; .07]							
SRMR	.05								.06							

HWB = happiness and well-being; PA = positive attitude; TQ = tranquility; JD = joy and desires; AN = absence of negativity; PD = personal development; PU = purpose; BL = belonging; LC = luck; for the item wordings see online materials Table B

#### Characteristics of the Final Versions of the CoDI Scales

We named the novel measures the “Complexity of Happiness Definitions and Intentions” (CoDI) Scales. The items can be found in the Appendix. Cronbach’s alpha for the subscales ranged between α = .63 and α = .87. To quantify associations, we used Pearson’s product-moment correlation coefficients. Descriptive statistics for the subscales and inter-factor correlations are displayed in Table 3.

HWB definitions-complexity and HWB-related intentions-complexity were approximately normally distributed. Correlations between acquiescence and the CoDI subscales, definitions-complexity, and intentions-complexity ranged between small and medium effect sizes (effect sizes in this study were interpreted in accordance with Cohen, 1988, where *r* > .30 indicates a medium effect size and *r* > .50 indicates a high effect size). As in Study 1, this indicates that people who tend to agree on opposite statements also tend to agree on items of the CoDI Scales. Although the general patterns of correlations were consistent between the unadjusted and adjusted versions of the scales, this result highlights the importance of taking response tendencies into account and that controlling for acquiescence might be a way to identify potential problems with response tendencies.

#### Nomological Net of the Novel Measures

To provide evidence for the convergent and discriminant validity of the novel measures, we examined bivariate correlations. HWB definitions-complexity and HWB-related intentions-complexity were positively associated (*r* = .48, *p* < .001). Correlations between corresponding scales for HWB definitions and HWB-related intentions showed that the corresponding scales are sufficiently associated but not so strong that we have to assume that the parallel versions capture the same construct. Further, these correlations suggest that endorsing a specific HWB definition and the intention to pursue a specific facet of HWB in everyday life differs in strength between different definitions and facets of HWB.

Next, we examined correlations with other measures. Overall, these correlational patterns correspond with the preregistered expectations, although they did not always reach the threshold of *r* >|.30| to provide evidence for meaningful associations (preregistered expectations and examined correlations are displayed in Table 4). Nevertheless, the correlational patterns of related scales clearly provide support for the convergent validity of the novel scales. For example, the Beliefs about Well-Being subscale pleasure showed the highest correlations with the CoDI subscales positive attitude and joy and desires, the subscale avoidance of negative showed the highest correlation with the CoDI subscale absence of negativity, the subscale self-development showed the highest correlation with the CoDI subscale personal development, and the subscale contribution to others showed the highest correlation with the CoDI subscale belonging. Generally, associations of HWB-related intentions with related scales tend to be stronger than of HWB definitions and reached more often the threshold of *r* >|.30|. Support for the discriminant validity appears to be provided for HWB definitions since the preregistered associations were mostly considerably stronger than other observed correlations that have not been preregistered. However, for HWB-related intentions, the discriminant validity is not always clear, since generally many associations could be observed between intentions and other measures that were not preregistered. Further, the nomological networks showed differences between subscales for positive attitude and tranquility as well as between subscales for personal development and purpose. As for the confirmatory factor analyses, these results suggest that it is reasonable to consider positive attitude and tranquility as well as personal development and purpose as separate factors.

#### Associations with Well-Being

For subjective well-being, the correlations with subscales for HWB definitions were small to moderate. Both positive (e.g., between personal development and positive affect: *r* = .28, *p* < .001) and negative (e.g., between absence of negativity and positive affect, *r* = -.16, *p* = .007) correlations were found. Definitions-complexity was only significantly correlated with positive affect (*r* = .13, *p* = .026). Subscales for HWB-related intentions were positively correlated with subjective well-being, and these correlations were generally higher than for subscales of HWB definitions (e.g., between positive attitude and positive affect: *r* = .55, *p* < .001). Intentions-complexity correlated significantly with all facets of subjective well-being (e.g., positive affect: *r* = .52, *p* < .001).

Associations of the CoDI Scales with psychological well-being showed similar correlation patterns. Only growth (*r* = .19, *p* = .002) and positive relations (*r* = .18, *p* = .003) correlated significantly with definitions-complexity and all facets correlated significantly with intentions-complexity (e.g., growth: *r* = .41, *p* < .001). In sum, associations with well-being were stronger and more consistent for HWB-related intentions and their complexity than for HWB definitions and their complexity. Further, these results indicate that the complexity of HWB definitions is positively associated with some facets of well-being and that the complexity of HWB-related intentions is positively associated with all investigated facets of well-being.

## General Discussion

We presented two studies in which we demonstrated that HWB-related intentions and their complexity were positively associated with subjective and psychological well-being. Associations between HWB definitions and well-being, however, were not always significant, in some cases negative and definitions-complexity was only positively related to positive affect, growth, and positive relations. The distributions of HWB definitions-complexity and of HWB-related intentions-complexity showed that people vary on a continuum on these characteristics and that people usually do not focus on one facet in their pursuit of HWB. The positive associations between these constructs and well-being demonstrated that for the experience of well-being, it does not only matter which specific facets of HWB people endorse but also the extent to which people define and pursue happiness in a multifaceted manner, which is in line with previous literature on similar questions (Grimm et al., 2015; Henderson & Knight, 2012; Huta & Ryan, 2010; Sirgy & Wu, 2009). However, since associations with well-being were more strongly and consistently for HWB-related intentions and their complexity than for HWB definitions and their complexity, the former emerged as more important for the experience of well-being than the latter.

To operationalize these constructs, we developed the CoDI Scales, a set of measures to assess HWB definitions, HWB-related intentions, HWB definitions-complexity, and HWB-related intentions-complexity. The scales distinguish eight facets of HWB: absence of negativity, positive attitude, tranquility, personal development, luck, joy and desires, purpose, and belonging. We provided evidence on the factorial structure, reliability, and validity of these measures. The CoDI Scales have several advantages compared to related scales. First, they cover eight different HWB definitions, whereas previous scales only cover a maximum of four different factors (e.g., McMahan & Estes, 2011b; Peterson et al., 2005). Hence, the CoDI Scales include HWB definitions that have previously not been included in similar scales, despite being important for lay people (e.g., tranquility or luck; Delle Fave et al., 2016; Oishi et al., 2013). Second, during the scale-development process, we considered a broad scope of focuses and perspectives on HWB definitions, including qualitative and quantitative studies of lay people, theoretical elaborations, and investigations of language use related to HWB in different cultures. Third, in contrast to previous scales, we took the distinction between HWB definitions and HWB-related intentions into account. The significance of this distinction is supported by our results, which showed that the two parallel scales measured related but not identical constructs. Fourth, the operationalization of HWB definitions-complexity and of HWB-related intentions-complexity allows the systematic investigation of these constructs in future research. In sum, the CoDI Scales are an extensive tool that allows a nuanced investigation of lay people's approach to HWB. For example, in a previous article using the CoDI Scales we showed that people who tend to be concerned about their level of happiness also tend to define HWB solely as the absence of negativity and to have no intentions to pursue any facets of HWB—not even the absence of negativity (Krasko et al., 2020).

Although it is well known that questionnaire-based results usually contain method variance that can be attributed to response tendencies like acquiescence (Danner et al., 2015; Soto et al., 2008; Wetzel et al., 2013), such response tendencies are rarely taken into account. In the present paper, we showed that acquiescence indeed affected the strengths of associations, although the general patterns of results remained unaffected. Response tendencies should much more frequently be considered in research that relies on self-report.

An important limitation of these studies is that we used German convenience samples, which limits the generalizability of the results. In particular, the results cannot be generalized to non-Western cultures since cultural differences in HWB definitions exist (Oishi et al., 2013; Uchida & Ogihara, 2012). Therefore, it would be an important next step to translate the scales into other languages and to investigate whether characteristics of the CoDI Scales as well as observed associations with well-being can be replicated in other cultures.

Moreover, future studies should investigate whether the factorial structure of the CoDI Scales can be confirmed in other samples since the results of our studies were rather exploratory and our decisions regarding the factor structure need further validation. For example, we decided to distinguish the factors positive attitude and tranquility as well as the factors personal development and purpose although this contradicts the results of the exploratory factor analyses. In both cases, the two pairs of factors represent HWB definitions that are related and thus could be expected to correlate with each other. In other words, positive attitude and tranquility both refer to positive emotions, whereas personal development and purpose both refer to eudaimonic definitions. Although this decision is supported theoretically as well as by the results, it needs to be re-evaluated in future studies.

Another limitation is the cross-sectional nature of the studies, which prohibits any causal conclusions between the variables of interest. Positive associations between lay definitions of HWB and actual levels of well-being have previously been reported (McMahan & Estes, 2011a, 2011b; Steger et al., 2008). Positive associations between constructs similar to the complexity of HWB definitions and actual levels of well-being have also previously been reported (Grimm et al., 2015; Henderson & Knight, 2012; Huta & Ryan, 2010). However, it remained unclear why these associations exist. It can be speculated that HWB definitions and their complexity are a prerequisite for HWB-related intentions and their complexity which, in turn, affect actual levels of well-being. Whether these links exist should be longitudinally investigated in the future.

Several open questions regarding HWB definitions and their complexity and HWB-related intentions and their complexity could become the subject of future research. For example, how can discrepancies between HWB definitions and HWB-related intentions be explained? It may be that such discrepancies can be explained by individual differences in personality, regulatory focus, or self-efficacy. Additionally, correlations showed that the extent of discrepancies differed between different HWB definitions, which might be attributable to differences in the characteristics of HWB definitions.

Results of the present studies as well as previous literature demonstrated that the diversity of a set of related constructs is predictive of outcomes over and above mean levels of these constructs (Huta & Ryan, 2010; Quoidbach et al., 2014; Sheldon & Niemiec, 2006). We suggest that the diversity of lay definitions might also be predictive of central outcomes in other areas of research on lay people’s views, like intelligence (Giraudeau et al., 2007), health (Bishop & Yardley, 2010), or quality of life in older age (Bowling & Gabriel, 2007). Our operationalization of definitions-complexity and intentions-complexity can be used in the future to investigate the diversity of lay definitions in other areas of research.

## Conclusion

We introduced the concepts complexity of HWB definitions (i.e., how many HWB definitions people endorse simultaneously) and complexity of HWB-related intentions (i.e., how many unique facets of HWB people intend to pursue in everyday life). These concepts can be operationalized with the CoDI Scales, a tool to assess peoples’ HWB definitions and their complexity as well as HWB-related intentions and their complexity. The CoDI Scales distinguish eight facets of HWB, including HWB definitions that have previously not been included in similar scales, despite being important for lay people (e.g., luck, tranquility). Further, we demonstrated that HWB-related intentions and their complexity were positively associated with well-being. For HWB definitions, relations with well-being were smaller and less consistent and definitions-complexity was only positively related to positive affect, growth, and positive relations. This paper highlights the importance of a multifaceted definition and pursuit of HWB for the actual levels of well-being and provides an extensive tool for a nuanced investigation of lay people's approach to HWB.

## Data Availability

Data files, analysis scripts (https://osf.io/5qy27/), and online supplementary materials (https://osf.io/gfytn/) are openly available on OSF.

## The CoDI Scales: German Version

Instruktion: Bitte geben Sie an, wie sehr die folgenden Aussagen auf Sie zutreffen. Antwortformat: 1 = trifft nicht zu, 2 = trifft kaum zu, 3 = trifft ein wenig zu, 4 = trifft etwas zu, 5 = trifft überwiegend zu, 6 = trifft voll und ganz zu.

**Table:**

	HWB Definitionen	HWB Intentionen	Subskala
	Itemstamm: Glück bedeutet für mich persönlich…	Itemstamm: Im Alltag versuche ich…	
1	sich im Hier und Jetzt auf das Positive zu konzentrieren	mich im Hier und Jetzt auf das Positive zu konzentrieren	Positive Einstellung
2	sllgemein eine positive emotionale Einstellung zum Leben zu haben	allgemein eine positive emotionale Einstellung zum Leben zu haben	Positive Einstellung
3	heiter durch das Leben zu gehen	heiter durch das Leben zu gehen	Positive Einstellung
4	ein friedliches Gemüt	ein friedliches Gemüt an den Tag zu legen	Innere Ruhe
5	Ruhe und Gelassenheit	ruhig und gelassen zu sein	Innere Ruhe
6	ein Gefühl der inneren Ruhe	ein Gefühl der inneren Ruhe zu haben	Innere Ruhe
7	das Ausmaß, in dem gegenwärtige Vorlieben erfüllt sind	meine gegenwärtigen Vorlieben zu erfüllen	Freude und Gelüste
8	ein großes Ausmaß an Vergnügen zu erleben	ein großes Ausmaß an Vergnügen zu erleben	Freude und Gelüste
9	gleichzeitige Befriedigung in mehreren Lebensbereichen	nach Befriedigung in mehreren Lebensbereichen gleichzeitig zu streben	Freude und Gelüste
10	dass man sich keine Sorgen macht	mir keine Sorgen zu machen	Abwesenheit von Negativem
11	sich nur selten schlecht zu fühlen	mich nur selten schlecht zu fühlen	Abwesenheit von Negativem
12	selten traurig zu sein	traurigen Ereignissen und Gedanken aus dem Weg zu gehen	Abwesenheit von Negativem
13	der Erwerb neuer Fähigkeiten	neue Fähigkeiten zu erwerben	Persönliche Entwicklung
14	Offenheit und Interesse für alle Fragen des Lebens zu zeigen	Offenheit und Interesse für alle Fragen des Lebens zu zeigen	Persönliche Entwicklung
15	über sich selbst hinauszuwachsen	über mich selbst hinauszuwachsen	Persönliche Entwicklung
16	einen klaren Sinn im Leben zu haben	einen klaren Sinn im Leben zu haben	Sinn
17	wegweisende Ziele im Leben zu haben, an denen man seine Handlungen ausrichten kann	wegweisende Ziele im Leben zu haben, an denen ich meine Handlungen ausrichten kann	Sinn
18	dass das eigene Leben eine verbleibende Bedeutung hat	meinem Leben eine verbleibende Bedeutung zu verleihen	Sinn
19	sich zugehörig zu fühlen	mich zugehörig zu fühlen	Zugehörigkeit
20	sich anderen nahe zu fühlen	mich anderen nahe zu fühlen	Zugehörigkeit
21	mit anderen in seiner Gemeinschaft verbunden zu sein	mit anderen in meiner Gemeinschaft verbunden zu sein	Zugehörigkeit
22	das Ergebnis von vorteilhaften Umständen	von vorteilhaften Umständen begünstigt zu sein	Zufallsglück
23	durch Glück oder Schicksal begünstigt zu sein	jemand zu sein, der durch Glück oder Schicksal begünstigt wird	Zufallsglück
24	zufällige Ereignisse, die einem einen Vorteil bringen	jemand zu sein, dem zufällige Ereignisse einen Vorteil bringen	Zufallsglück

## The CoDI Scales: English Version

Instruction: Please indicate to what extent you agree with the following statements. Response format: 1 = does not apply, 2 = applies hardly, 3 = applies a little, 4 = applies somewhat, 5 = applies strongly, 6 = applies completely.

**Table:**

	Hwb definitions	Hwb intentions	Subscale
	Item stem: for me personally, happiness means…	Item stem: in daily life, i try…	
1	to focus on the positive aspects in the here and now	to focus on the positive aspects in the here and now	Positive attitude
2	to have a positive attitude towards life in general	to have a positive attitude towards life in general	Positive attitude
3	to go through life cheerfully	to go through life cheerfully	Positive attitude
4	a peaceful state of mind	to have a peaceful state of mind	Tranquility
5	calmness and tranquility	to be calm and tranquil	Tranquility
6	a feeling of inner quietness	to have a feeling of inner quietness	Tranquility
7	the extent to which current preferences are satisfied	to satisfy my current preferences	Joy and desires
8	experiencing a great deal of pleasure	to experience a great deal of pleasure	Joy and desires
9	simultaneous satisfaction in several areas of life	to strive for simultaneous satisfaction in several areas of life	Joy and desires
10	not to worry	not to worry	Absence of negativity
11	to rarely feel bad	to rarely feel bad	Absence of negativity
12	to rarely be sad	to avoid sad experiences and thoughts	Absence of negativity
13	the acquisition of new skills	to acquire new skills	Personal development
14	to show openness and interest for all the questions about life itself	to show openness and interest for all the questions about life itself	Personal development
15	to develop beyond oneself	to develop beyond myself	Personal development
16	to have a clear meaning in life	to have a clear meaning in life	Purpose
17	to have guiding goals in life that direct one's own actions	to have guiding goals in life that direct my own actions	Purpose
18	that one’s own life has a lasting meaning	that my own life has a lasting meaning	Purpose
19	to feel a sense of belonging	to feel a sense of belonging	Belonging
20	to feel close to others	to feel close to others	Belonging
21	to be connected with others in one’s community	to be connected with others in my community	Belonging
22	the result of advantageous circumstances	to be favored by advantageous circumstances	Luck
23	to be favored by luck or fate	to be someone who is favored by luck or fate	Luck
24	to benefit from random events	to be someone who benefits from random events	Luck

## Footnotes

1 The preregistration, deviations from the preregistration (https://osf.io/bq4hr & https://osf.io/pwxcs/), and all data files and R-codes (https://osf.io/5qy27/) are available at OSF.

2 Prior to the analyses, we contrasted different ways of calculating complexity, which we share in the online materials (https://osf.io/gfytn/; file “Ideas for computing complexity.xlsx”).

3 The online materials are available at OSF: https://osf.io/gfytn/.

4 The unadjusted and adjusted item distributions can be found in the online materials at OSF: https://osf.io/gfytn/.

5 The preregistration, deviations from the preregistration (https://osf.io/jcb75 & https://osf.io/pwxcs/), and all data files and R-codes (https://osf.io/5qy27/) are available at OSF.

6 The unadjusted and adjusted item distributions can be found in the online materials at OSF: https://osf.io/gfytn/.

7 The online materials are available at OSF: https://osf.io/gfytn/.

8 We performed robustness checks to investigate whether dropping items and allowing correlations between items affect the results of the validation analyses. Specifically, we investigated correlations between the final scales with three items for each factor and the initial long version of the scales. Further, we investigated correlations between the manifest (final) scale scores and the latent factor scores of the model with correlated residuals (online materials Table C). The correlation patterns between corresponding scales were always very high (≥ .88). Therefore, scale meanings did not appear to be affected by decisions in the scale development. Further, we recalculated the validation analyses using the latent factor scores (online materials Table D). These additional analyses show that the model specifications did not fundamentally affect the results of this paper and the subsequent conclusions.

## Declarations

### Preregistration

The studies were preregistered on OSF: https://osf.io/bq4hr & https://osf.io/jcb75
